# Selective Depletion of rRNA Enables Whole Transcriptome Profiling of Archival Fixed Tissue

**DOI:** 10.1371/journal.pone.0042882

**Published:** 2012-08-10

**Authors:** John D. Morlan, Kunbin Qu, Dominick V. Sinicropi

**Affiliations:** Genomic Health Inc., Redwood City, California, United States of America; University of Connecticut Health Center, United States of America

## Abstract

We report a method for Selective Depletion of abundant RNA (SDRNA) species from Human total RNA isolated from formalin-fixed, paraffin-embedded (FFPE) tissue, here demonstrating removal of ribosomal and mitochondrial transcripts from clinical FFPE tissue RNA archived up to 20 years. Importantly, SDRNA removes 98% of targeted RNAs while preserving relative abundance profiles of non-targeted RNAs, enabling routine whole transcriptome analysis of clinically valuable archival tissue specimens by Next-Generation Sequencing.

## Introduction

The large repositories of FFPE archival tumor specimens linked to long term clinical records provide a rich resource for discovery of prognostic and predictive biomarkers. Whole-transcriptome analysis by Next-Generation Sequencing (NGS) technologies provides an opportunity to dramatically expand the number of biomarkers that can be surveyed in these specimens [1]–[2]. To date, relatively few NGS studies of fixed archival tissue specimens have been reported [3]–[5]. Efficient whole transcriptome analysis requires the selective removal of ribosomal RNA (rRNA) which can account for as much as 85% of total RNA. The most commonly employed enrichment methods, namely, polyA+ selection or solid-phase capture and removal, are expensive and cumbersome or are ineffective with the highly fragmented RNA that exists in FFPE tissue. PolyA+ selection has the further disadvantages that coverage is limited to the 3′ends of transcripts [5] and that non-polyadenylated transcripts are not captured in the final library [6]. Methods that employ pseudo-random cDNA priming to avoid ribosomal sequences [7] have been reported to have poor start-site complexity compared to other methods [8]. Methods that use non-specific degradation of highly-abundant transcripts [9] (see also Illumina Application Note #15014673 Rev. C) are technically cumbersome to perform and difficult to reproduce.

We describe here a new method that we call selective depletion of abundant RNA (SDRNA). Short (50–80 bases) antisense DNA probes are constructed that are complementary to and tile across the entire length of sequences targeted for removal. These sequences form RNA:DNA hybrids across the entire length of the targeted RNA species, including species that are fragmented. Treatment with RNaseH followed by DNaseI effectively destroys the targeted RNA species as well as residual DNA probes. This process can be easily reconfigured to target different RNA species, preserves the integrity of non-targeted RNAs, can be performed in about an hour and the resulting depleted RNA can be used as input to virtually any cDNA library construction method.

## Results

Human cells synthesize rRNAs as single 13 kb transcripts [10] (Figure S1a). We chose to target only the 18S, 5.8S and 28S rRNA genes; the intervening portion of this transcript is hereafter referred to as non-targeted rRNA. SDRNA version 1 (SDRNA1) uses probes targeting 18S and 28S rRNA (Table S1) while SDRNA version 2 (SDRNA2) includes additional probes (Table S2) targeting 5.8S rRNA as well as 12S and 16S rRNAs (mtrRNA, Figure S1b). To evaluate the efficiency of rRNA depletion we prepared undepleted libraries as well as SDRNA1 and SDRNA2 libraries using as starting material high-quality, intact RNA from fresh-frozen (FF) tissue and also a pool of FFPE tumor tissue RNA (see Methods). Libraries were sequenced using the Illumina GAIIx or HiSeq platform. A polyA+ library from the high-quality RNA sample was also prepared and sequenced for comparison. We did not evaluate polyA+ selection of FFPE RNA because it captures only the 3′-UTRs of polyadenlylated transcripts [5]. Total yield of reads (21–29 million for GAII, 52–74 million for HiSeq) and percentages of uniquely-mapping reads (68%–77% for GAII, 60%–79% for HiSeq) were comparable across all libraries used for these comparisons (Libraries 1–8, Table S3).

**Figure 1:**
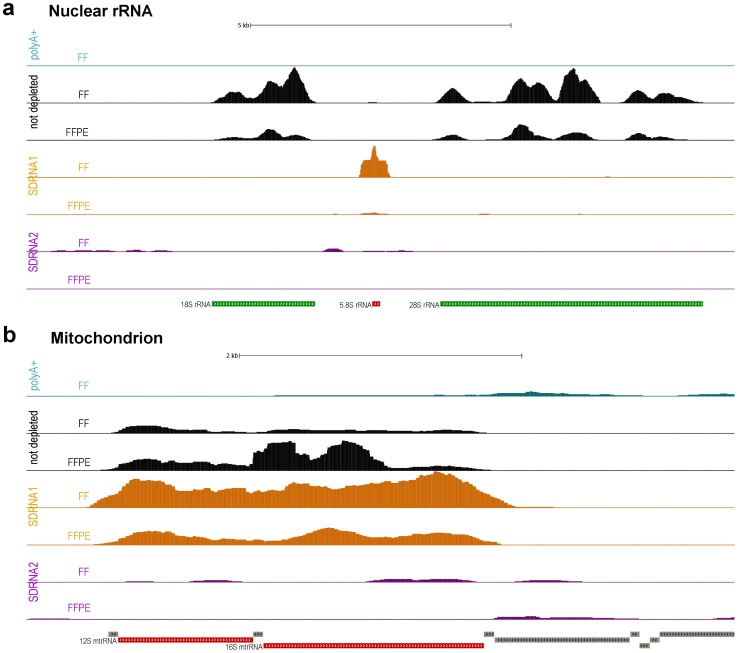
Read density of targeted regions. Read density plotted across regions targeted for depletion and compared between different depletion methods. Regions targeted for depletion (SDRNA1 = green, SDRNA2 = red and green) are indicated at the bottom of each figure. Density values were computed using igvtools count (www.broadinstitute.org/igv/igvtools) and plotted using UCSC’s Genome Browser. FF – Fresh-frozen; FFPE – Formalin-fixed, paraffin-embedded.(a) Reads are mapped to chrUn_gl000220, an unplaced genomic contig from hg19 containing the 13 kb rRNA transcript described in Supplementary Figure 1a. (b) Reads are mapped to the human mitochondrion from hg18 (Supplementary Figure 1b).

Figure 1 graphically displays read coverage for targeted rRNA and mtrRNA regions in these library preparations. Consistent with its design, SDRNA1 effectively removed 18S and 28S rRNAs, but not 5.8s rRNA or mtrRNAs, in libraries made from either intact or FFPE RNA. The 5.8S rRNA is observed to increase in abundance in the SDRNA1 library prepared from intact RNA which may be a consequence of having depleted the more abundant 18S and 28S species in this library. PolyA+ selection or SDRNA2 treatment both significantly reduced the abundance of reads mapping to all rRNAs including 5.8S rRNA and the 12S and 16S mtrRNAs for both intact and FFPE RNA libraries. In the SDRNA2 library prepared from intact RNA, targeted rRNAs (18S, 28S, 5.8S rRNAs and 12S and 16S mtrRNAs) account for less than one percent of uniquely-mapped reads (Table S3). The sum of all rRNAs (targeted and untargeted regions) was just over six percent in the SDRNA2 library made from intact RNA, essentially identical to that obtained from a polyA+ library prepared from the same source RNA (Table 1). These results demonstrate that depleting the targeted regions shown in Figure 1 is sufficient to remove the vast majority of ribosomal and mitochondrial RNAs from Human total RNA. The SDRNA2 library prepared from FFPE RNA likewise exhibits a dramatic reduction in the proportion of total rRNA reads compared to an undepleted library (Table 1).

**Table 1:** Proportion of reads uniquely-mapping to rRNA or non-rRNA categories.

UniqueLibrary ID	Tissue Treatment	Depletion Method	non-rRNA	rRNA
Library_01	FFPE	SDRNA1	84.1%	15.9%
Library_02	FFPE	untreated	39.6%	60.5%
Library_03	FFPE	SDRNA2	98.1%	1.9%
Library_04	FFPE	SDRNA1	88.4%	11.6%
Library_05	FF	polyA+	94.4%	5.5%
Library_06	FF	SDRNA1	64.8%	35.2%
Library_07	FF	SDRNA2	93.8%	6.2%
Library_08	FF	untreated	5.6%	94.4%

The rRNA category represents all rRNAs encoded in both nuclear and mitochondrial genomes (see Supplementary Figure 1). Non-rRNA includes all other uniquely-mapping reads.

**Figure 2:**
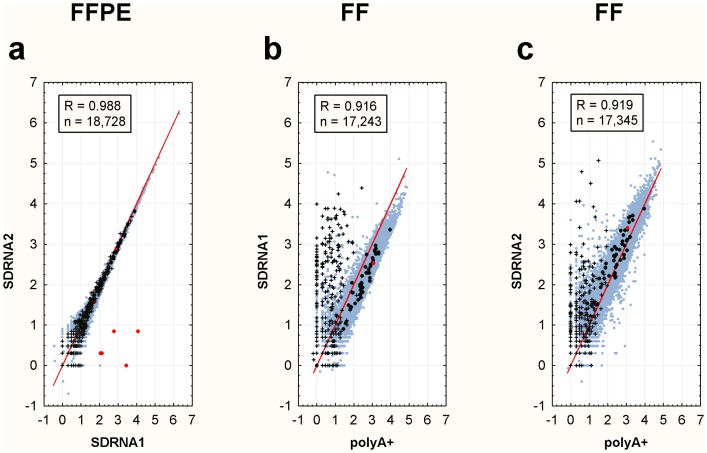
Scatterplots of RefSeq transcript abundance. Log_10_ read counts are plotted. Transcripts with zero reads are excluded from analysis. An identity line is shown in each plot for reference. Sub-populations are indicated as follows: Black cross – sno-, sc-, sn- and mir-RNAs from RefSeq; Black and red circles – Genes homologous to SDRNA and SDRNA2 probes, respectively (Table S4); Light blue circles – All other RefSeq transcripts. FF – Fresh-frozen; FFPE – Formalin-fixed, paraffin-embedded. (**a**) FFPE RNA Pool: SDRNA1 vs SDRNA2. (**b**) FF breast RNA : polyA+ vs SDRNA1. (**c**) FF breast RNA : polyA+ vs SDRNA2.

**Figure 3:**
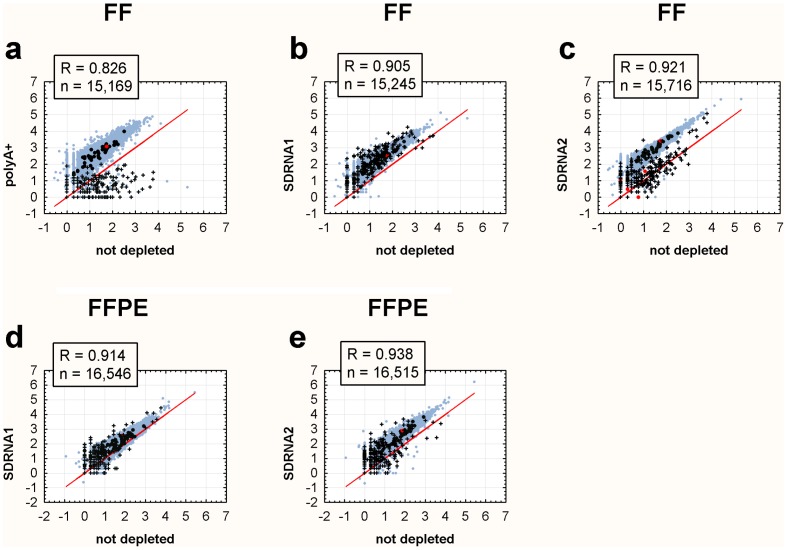
Scatterplots of RefSeq transcript abundance: Depleted vs. Undepleted. Log_10_ read counts are plotted. Transcripts with zero reads are excluded from analysis. An identity line is shown in each plot for reference. Sub-populations are indicated as follows: Black cross – sno-, sc-, sn- and mir-RNAs from RefSeq; Black and red circles – Genes homologous to SDRNA and SDRNA2 probes, respectively (Table S4); Light blue circles – All other RefSeq transcripts. FF – Fresh-frozen; FFPE – Formalin-fixed, paraffin-embedded. (**a**) FF Breast RNA: not depleted vs polyA+ (**b**) FF Breast RNA: not depleted vs SDRNA1 and (**c**) FF Breast RNA: not depleted vs SDRNA2. (**d**) FFPE RNA pool: not depleted vs SDRNA1 and (**e**) FFPE RNA pool: not depleted vs SDRNA2.

Transcript abundance was highly reproducible in technical replicates both within SDRNA method (SDRNA1 R = 0.96, SDRNA2 R = 0.95) and between SDRNA method (R = 0.99, Figure 2a). SDRNA libraries exhibit Pearson R correlations >0.9 when compared to polyA+ libraries (Figure 2b,c) or undepleted libraries (Figure 3) prepared from the same RNA. Correlations between SDRNA and undepleted libraries (Figure 3b, c) are at least as good as the correlation between the polyA+ and undepleted library (Figure 3a).

Pearson correlations across method (SDRNA vs polyA+) are lower than within method (SDRNA vs SDRNA). Inspection of the outlier data points in the polyA+ versus SDRNA scatter plots shown in Figure 2b and 2c reveal the majority are annotated as small nuclear (snRNA), small nucleolar (snoRNA), small cytoplasmic (scRNA) or micro (mirRNA) RNAs which demonstrates that these transcripts are under-represented in the polyA+ library as compared to SDRNA1 or SDRNA2. This result is consistent with other studies comparing polyA+- and non-polyA+-based depletion methods [6]–[7]. A survey of all transcripts annotated by the UCSC Genome Browser or ENSEMBLE database as sn-, sc-, sno- or mir-RNAs reveals substantial enrichment in SDRNA2 in most of these categories compared to polyA+ (Figure S2).

Based on RefSeq annotations 89.9% and 89.2% of transcripts are represented by at least one read in the SDRNA1 and SDRNA2 libraries made from the FFPE RNA pool on the HiSeq platform, respectively, and approximately 83% of either the polyA+ or SDRNA1 library prepared from intact RNA on the GAIIx platform are represented by at least one read, demonstrating that most transcripts are being detected regardless of method. Further, of 4,283 RefSeq genes not detected by SDRNA1 in intact RNA, 3,591, or 84%, were likewise not detected in the polyA+ library. The remaining 692 transcripts not reported in SDRNA1 are low-abundance as measured in the polyA+ library (<35 reads/gene). More transcripts appear to have been detected in the SDRNA2 library than the polyA+library but this is likely due to differences in the depth of sequencing (compare libraries 5 and 7, Table S3). These data suggest that SDRNA is specific for its intended targets. To further investigate the effects of SDRNA on non-targeted transcripts all probes (Tables S1, S2) were subjected to low-stringency BLAST analysis (see Methods) against Human RefSeq transcripts. Forty-six of the 122 probes have at least 50% nucleotide identity with 66 individual genes (Table S4). These genes, categorized by their homology to all SDRNA or SDRNA2-only probes, are highlighted in Figures 2 and 3. The majority of these genes track along the identity line in scatterplots comparing different methods (Figures 2 and 3). Only seven of these genes, all belonging to the MTRNR2-like family of genes, were adversely affected by treatment with SDRNA2 (Figure 2a, red circles), as might be expected due to the extensive sequence homology of this gene family with mitochondrial 16S rRNA, one of the targets of SDRNA2.

Expression of tumor-specific markers was investigated by RNA-Seq using SDRNA1 libraries in a cohort of twelve tumor and twelve unmatched normal breast cancer FFPE biopsy specimens. The archive age of each specimen was 10–12 years post fixation and embedding. Average within-tumor and within-normal expression values were computed for each gene and log tumor/normal fold-changes computed from the RNA-Seq data. Likewise, average log tumor/normal fold-changes were computed for 43 genes using available RT-PCR assays. Pearson correlations between RNA-Seq and relative qRT-PCR for the forty-three genes were greater than 0.9 over a 10 log range of expression fold-change between the tumor and normal specimens (Figure S3).

## Discussion

**Figure 4:**
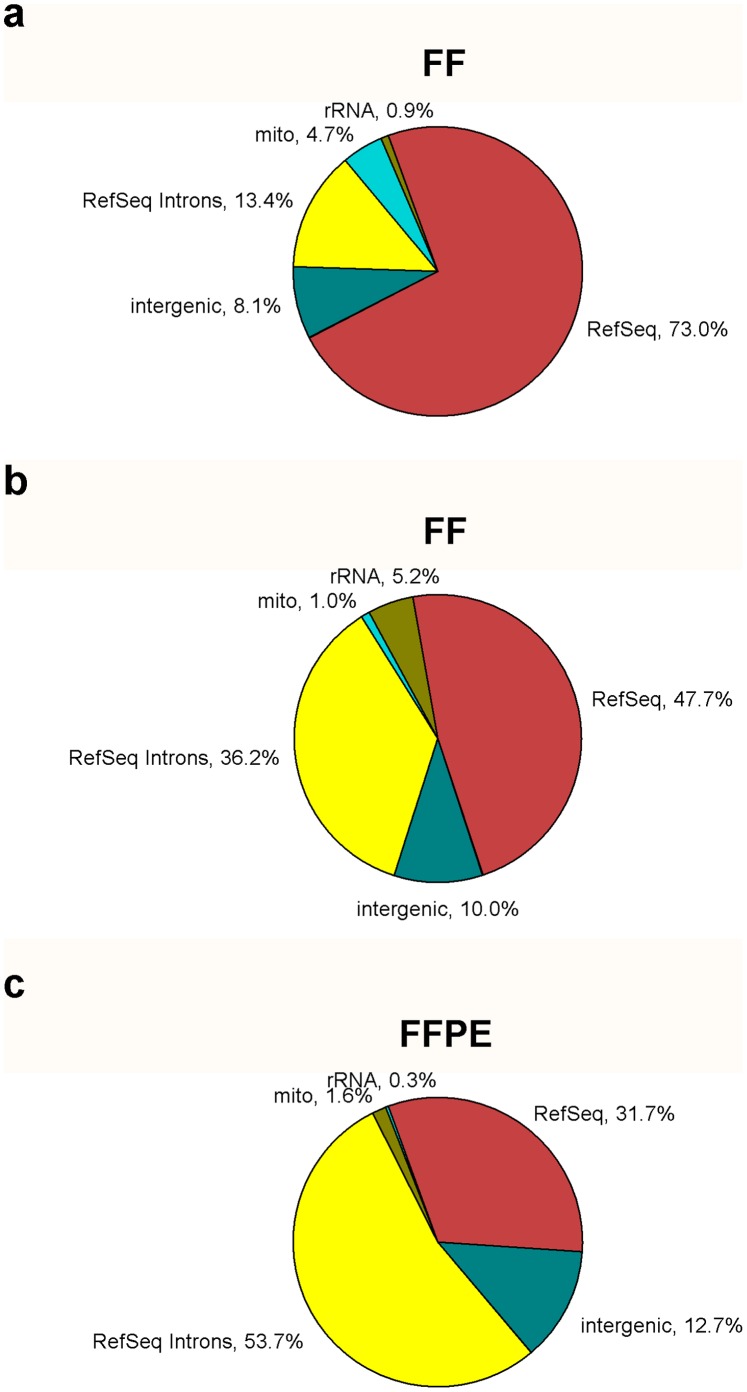
Distribution of uniquely-mapped reads across broad RNA classes. rRNA class includes all reads mapping to the 13 kb transcript shown in Supplementary **Figure 1a** **.** Mito class refers to all reads mapping to the mitochondrial genome shown in Supplementary Figure 1b. The Intergenic class encompasses all remaining reads not mapping to RefSeq annotations or their associated introns. FF – Fresh-frozen; FFPE – Formalin-fixed, paraffin-embedded. (a) polyA+ FF breast RNA.(b) SDRNA2 FF breast RNA. (c) SDRNA2 FFPE RNA pool.

The results described here provide evidence that transcript abundances can be reliably measured in RNAs extracted as long as twelve years after fixation and embedding. The removal of abundant rRNAs facilitates these measurements by increasing the proportion of reads mapping to non-rRNA categories of expressed transcripts. Only about 2.4% of reads map to annotated RefSeq exons from an undepleted library prepared from the intact RNA used in the present study (Library 8, Table S3). This value increases to 73% in the polyA+ library and 48% in the SDRNA2 library (Figure 4a and b). Fewer reads map to exons in the SDRNA2 library due to an increase in reads mapping to introns, from ∼13% in the polyA+ library to 36% in the SDRNA2 library. (Figure 4a and b). We speculate that some fraction of incompletely-transcribed mRNAs will be partially-spliced or un-spliced but not polyadenylated [11]. These partial transcripts should be retained in RNA extracts following SDRNA, but not polyA+, treatment. Spliced but undegraded introns may also contribute to this phenomenon. Our result is also consistent with other studies comparing polyA+ with non-polyA+ depletion methods [12].

In libraries made with the FFPE RNA pool used in the current study about 10% of reads map to annotated RefSeq exons when the library is not depleted (Library 2, Table S3). This value increases to about 32% in the SDRNA2 library (Figure 4c) and the proportion of reads contributed by introns (∼54% ) is even greater than in libraries prepared from intact RNA (compare Figure 4c with Figure 4a, b). These higher intronic read fractions have been observed for the majority of SDRNA2 libraries prepared from FFPE RNA in our laboratory and are very similar to results reported from others who have prepared libraries from nuclear RNA [13]. A possible explanation for the increased proportion of intronic reads from FFPE specimens may be that the nuclear envelope, which remains intact in fixed tissues, may protect against degradation by RNase in the cytoplasm during formalin fixation.

In addition to the results reported in this paper we have used SDRNA2 in the preparation of more than two-hundred RNA-Seq libraries. The sources of input RNAs have been mainly FFPE breast tissue biopsy specimens with archive ages ranging from 5–20 years but also include more recent tumor and normal specimens from colon, renal and prostate tissues. The fraction of reads mapping to mitochondrial 12S and 16S mtrRNAs is less than 1% of mapped reads on average; the 18S, 28S and 5.8S rRNA species taken together average less than 2% of mapped reads. We have reported the results of experiments using both SDRNA1 and SDRNA2 to illustrate the ease with which the method can be re-configured to target different RNA species; however, we have adopted SDRNA2 as our standard depletion method. SDRNA currently works well on total RNA inputs as low as fifty nanograms and lower inputs are being investigated. In our laboratory SDRNA is routinely used with two very different library preparation chemistries: Targeted rRNAs average 1% when using SDRNA2 with the Epicentre’s non-ligation ScriptSeq ™ Kit as compared with 1.8% for Illumina’s ligation-based RNA-Seq Kit.

Since the development and implementation of SDRNA in our laboratory, Epicentre has released RiboZero™, currently the only commercially-available method that can effectively deplete rRNA species from fragmented total RNA. In our laboratory SDRNA and RiboZero™ are very comparable with respect to the magnitude of depletion and hands-on time (data not shown). However, SDRNA currently costs less than RiboZero™ on a per sample basis and can be readily re-configured to target additional RNA species if the need should arise. In summary, SDRNA is inexpensive, uses materials and reagents common to molecular biology laboratories, can be completed in less than an hour and is amenable to high-throughput sample processing and robotic automation. We believe SDRNA will have broad applications in addition to expression profiling, such as discovery of novel non-coding RNAs, splice isoforms, fusion transcripts and somatic mutations in total RNA from archival clinical specimens.

## Materials and Methods

### Ethics Statement

All samples were obtained according to a protocol approved by Essex Institutional Review Board (Lebanon, NJ). The requirement for informed consent was waived as all samples were de-identified. The protocol was approved by expedited review under 45 CFR 46.110 authority.

### Patient Samples

Normal and tumor FFPE breast biopsy specimens were obtained from Marin General Hospital (Greenbrae, CA). Except as noted above, all other FFPE tissues used for studies reported in this paper were obtained from patient breast tumor biopsy samples submitted to Genomic Health®, Inc. for use in the onco*type*DX® Breast Cancer Assay.

### RNA Extraction and Intact RNA

RNAs were extracted using a High Pure miRNA Kit™ (Roche, Mannheim, Germany). RNA was extracted from the tumor specimens approximately ten years post-fixation; some normal specimens were extracted approximately three months post-fixation and others approximately ten years post-fixation. High-quality, intact human mammary gland total RNA was obtained from Clontech (Mt. View, CA, catalog# 636576).

An FFPE Breast Tumor Tissue RNA Pool was created by mixing in equal portions RNAs extracted from multiple breast tumor biopsy samples submitted to Genomic Health®, Inc. for use in the onco*type*DX® Breast Cancer Assay. These samples were extracted less than one year post-fixation using the MasterPure™ Kit (Epicentre Biotechnologies).

### SDRNA Depletion of RNA

SDRNA method 1 uses eighty-eight non-overlapping synthetic DNA probes (Integrated DNA Technologies, Coralville, IA, see Table S1), 56 to 80 bases in length and representing the entire complementary sequences of human 18S rRNA (GenBank accession M10098) and 28S rRNA (GenBank accession M11167) at a final concentration of 0.5 µM for each probe (44 µM total). SDRNA method 2 uses the DNA probes of method 1 at 0.5 µM each plus twelve probes targeting 12S mtrRNA (GeneID 4549), twenty probes targeting 16S mtrRNA (GeneID 4550), and two probes targeting 5.8S rRNA (NR_003285) at 0.05 µM each (Table S2). One microliter of either the SDRNA1 or SDRNA2 pool was mixed with 50 nanograms to 1 microgram of total or amplified RNA in a final volume of 5 µL 1x Hybridization Buffer (100 mM Tris-HCl, 200 mM NaCl). The mixture was heated to 95°C for 2 minutes, then slow-cooled to 22°C (0.1°C/s), incubated an additional 5 minutes at 22°C, and placed on ice. Ten units of Hybridase™, a thermostable RNAseH (Epicentre, Madison, WI), was added along with 1 µL of 10x RNAseH digestion buffer (500 mM Tris-HCl, 1 M NaCl, 200 mM MgCl_2_) in a final reaction volume of 10 µL, incubated at 37°C for thirty minutes and placed on ice. DNA probes were removed by DNAse treatment using an RNAse-free DNAse Kit (QIAGEN, Valencia, CA) in a final volume of 100 µL according to manufacturer’s instructions and then purified using a MinElute RNEasy Kit (QIAGEN). RNA yield was determined by a RiboGreen assay (Invitrogen, Carlsbad, CA) according to manufacturer’s instructions. Depletion of one microgram of total RNA typically yields 90–130 ng of depleted RNA. The entire process takes approximately one hour to complete.

### RNA-Seq Libraries Prepared from Total RNA – Illumina® Protocol

The residual RNA following depletion of total RNA was used as input to prepare sequencing libraries. Libraries were prepared according to the mRNA Sequencing Sample Preparation Guide (part# 1004898 Rev. D, Illumina®, San Diego, CA) and using the mRNA-Seq Kit™ (Illumina®, San Diego, CA) with the following modifications: When using RNA derived from FFPE tissue the fragmentation and polyA+ selection steps were omitted. Random hexamers and enzyme reagents were obtained from New England Biolabs (Ipswich, MA); adapter oligos and PCR primers were obtained from Integrated DNA Technologies (Coralville, IA); in certain instances a Pippen Prep™ (Sage Science, Beverly, MA) was substituted for conventional agarose-gel electrophoresis as the method of size-selection.

### RNA-Seq Libraries Prepared from Total RNA – ScriptSeq™ Protocol

The residual RNA following depletion of total RNA was used as input to prepare strand-specific cDNA libraries. Libraries were prepared using the ScriptSeq™ mRNA-Seq Library Preparation Kit (Illumina compatible version, Epicentre, Madison, WI) following the manufacturer’s instructions.

### Sequencing

All libraries reported in this study were sequenced on an Illumina GAIIx or HiSeq2000 platform and used Illumina’s version 4 or TruSeq sequencing chemistry. Each library was loaded in one lane of a Single Read v4 or HiSeq flow cell (Illumina) at a concentration of 6 pM. Unless otherwise noted all libraries were sequenced to 50–51 cycles in one direction.

### PCR and RT-PCR

Reverse Transcription was performed using an OmniScript™ RT kit (QIAGEN) according to manufacturer’s instructions and using gene-specific primers to interrogate genes of interest. PCR was performed on an ABI 7900HT using TaqMan® Universal PCR Master Mix, No Amperase® UNG (Life Technologies, Carlsbad, CA) or on a LightCycler 480 (Roche Applied Science, Indianapolis, IN) using QuantiTect Probes PCR Master Mix™ (QIAGEN). In both cases 100 nM each primer and 450 nM probe was used in a 5 µl reaction volume. All PCR and RT-PCR assays were developed and validated at Genomic Health, Inc.

### Data Analysis and Processing

#### Adapter Trimming

Adapter length varies due to the broad distribution of fragment sizes. A trimming procedure was applied to ensure maximized trimming of the adapter contents from each read using the ShortRead package from Bioconductor (http://www.bioconductor.org/). Full length of the adapter sequence was shortened one base at a time and aligned to the 3′-end of each read with varied mismatch tolerance to determine the length of sequence to be removed. This resulted in the removal of six bases from the 5′- end and eight bases from the 3′-end of all reads.

#### Read Mapping

Reads were mapped to Human Genome version 18 and later 19 (UCSC). CASAVA (version 1.6 and 1.7), the standard data analysis pipeline from Illumina, utilizes “contaminated sequences” to screen out reads mapped to sequences which include the whole 43 KB of Human ribosomal DNA complete repeating unit (HSU13369), Illumina adapter sequences and Illumina PhiX control sequences. Mapping to the above listed reference sequences was also performed using Bowtie (http://bowtie-bio.sourceforge.net/index.shtml). Mapping rates did not differ significantly between the two algorithms. Only uniquely mapped reads were further analyzed and reported. A Perl script was implemented to bin reads mapped to the whole ribosomal RNA into 18S, 28S, 5.8S and non-targeted rRNA categories. Gene count data for this study has been deposited in the Dryad depository: http://dx.doi.org/10.5061/dryad.k83b8.

### BLAST Analysis

BLAST (http://blast.ncbi.nlm.nih.gov/) was performed with all probe sequences listed in Tables S1 and S2 against Homo sapiens refseq_rna using the blastn algorithm (expect = 10, word size = 11, Match score = 2, Mismatch score = −3, Gap cost = 5, Gap extension cost = 2, filter low complexity regions), automatically adjusted for short input sequences.

## Supporting Information

Figure S1 **Regions targeted for depletion by SDRNA.** Adjacent, non-overlapping anti-sense DNA oligonucleotides (black bars, see Tables S1 and S2) were designed against the highlighted sequences. Regions targeted for depletion by SDRNA1 (see text) are highlighted in green. Regions targeted for depletion by SDRNA2 combine probes from regions highlighted in red and green. (**a**) Human ribosomal RNA complete repeating unit (GenBank accession U13369.1) showing bases 1–13,000. Twenty-four 80-mers and one 56-mer were designed against the 18S rRNA. Sixty-two 80-mers and one 65-mer were designed against the 28S rRNA. Two 78-mers were designed against the 5.8S rRNA. (**b**) Human mitochondrial genome (NCBI reference sequence NC_012920.1) showing bases 1–5,000. Eleven 80-mers and one 74-mer were designed against MTRNR1 (12S rRNA). Nineteen 80-mers and one 39-mer were designed against MTRNR2 (16S rRNA).
(TIF)

Figure S2 **Enrichment of ncRNA transcripts in SDRNA libraries.** Comparing FF breast depleted with SDRNA2 or polyA+ selection. Classes are based on UCSC Genome Browser annotations for sno/miRNAs. 7SK coordinates are from ENSEMBLE.
(TIF)

Figure S3 **Cross-platform correlation of RefSeq transcript abundances.** Twelve FFPE tumor tissue and twelve FFPE normal tissue breast cancer libraries were prepared using SDRNA1 and analyzed in sixteen lanes on two flowcells using indexing. Read abundance was averaged on each flowcell for each gene across all six tumor libraries and likewise across all six normal libraries. Read counts less than ten were excluded. Log_2_ Tumor/Normal fold-changes for forty-three genes are plotted between RNA-Seq data (x-axis) and TaqMan® RT-PCR data (y-axis). Scatterplots of average tumor/normal expression from (**a**) flowcell 1 and (**b**) flowcell 2.
(TIF)

Table S1 **DNA probes used for SDRNA1 and SDRNA2.**
(XLSX)

Table S2 **DNA probes used for SDRNA2 only.**
(XLSX)

Table S3 **Summary of read level and gene level data.** Read lengths following adapter trimming are reported. FFPE - formalin-fixed, paraffin-embedded. FF - fresh frozen. Read level data is reported as a percentage of uniquely-mapped reads. SDRNA and SDRNA2 only homologs – genes homologous to SDRNA or SDRNA2 probes based on BLAST.
(XLSX)

Table S4 **SDRNA probes with homology to RefSeq genes.** Probes were BLASTed against Human RefSeq. Probes with at least 50% nucleotide identity are listed.
(XLSX)
