# Supplementary figures and images for: Selective Depletion of rRNA Enables Whole Transcriptome Profiling of Archival Fixed Tissue
Source: PLoS One. 2012 Aug 10;7(8):e42882. doi: 10.1371/journal.pone.0042882 (PMC3416766; doi:10.1371/journal.pone.0042882)

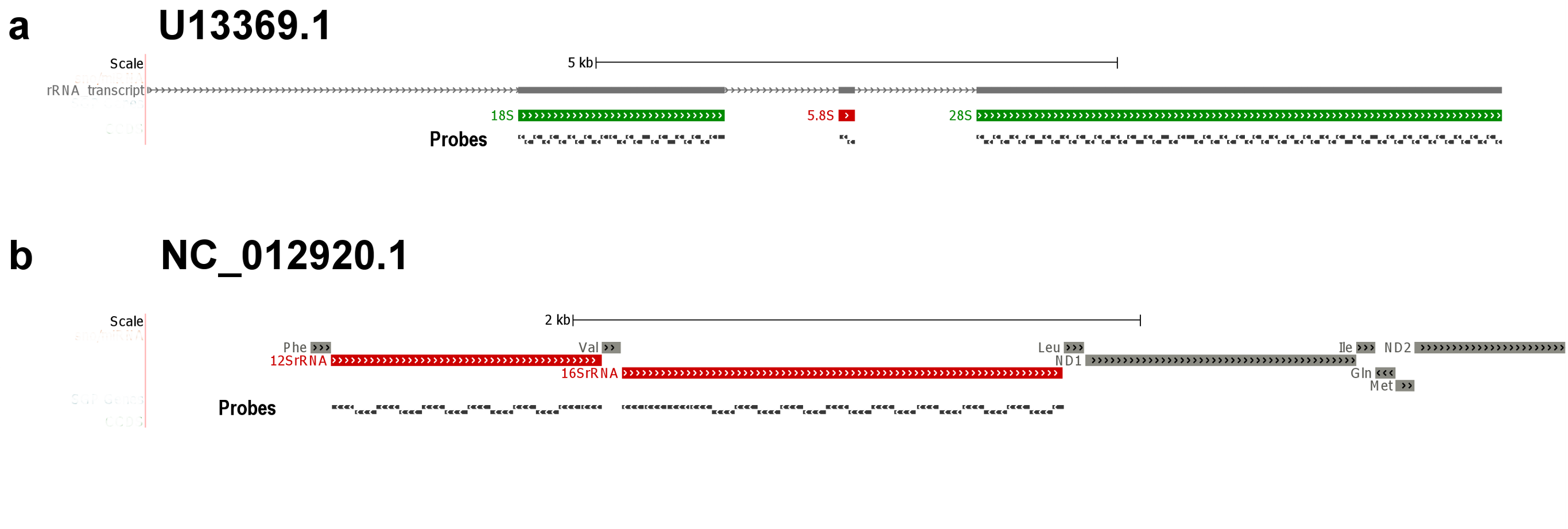

Supplement: Figure S1 — Regions targeted for depletion by SDRNA. Adjacent, non-overlapping anti-sense DNA oligonucleotides (black bars, see Tables S1 and S2) were designed against the highlighted sequences. Regions targeted for depletion by SDRNA1 (see text) are highlighted in green. Regions targeted for depletion by SDRNA2 combine probes from regions highlighted in red and green. (a) Human ribosomal RNA complete repeating unit (GenBank accession U13369.1) showing bases 1–13,000. Twenty-four 80-mers and one 56-mer were designed against the 18S rRNA. Sixty-two 80-mers and one 65-mer were designed against the 28S rRNA. Two 78-mers were designed against the 5.8S rRNA. (b) Human mitochondrial genome (NCBI reference sequence NC_012920.1) showing bases 1–5,000. Eleven 80-mers and one 74-mer were designed against MTRNR1 (12S rRNA). Nineteen 80-mers and one 39-mer were designed against MTRNR2 (16S rRNA). (TIF) [file pone.0042882.s001.tif]

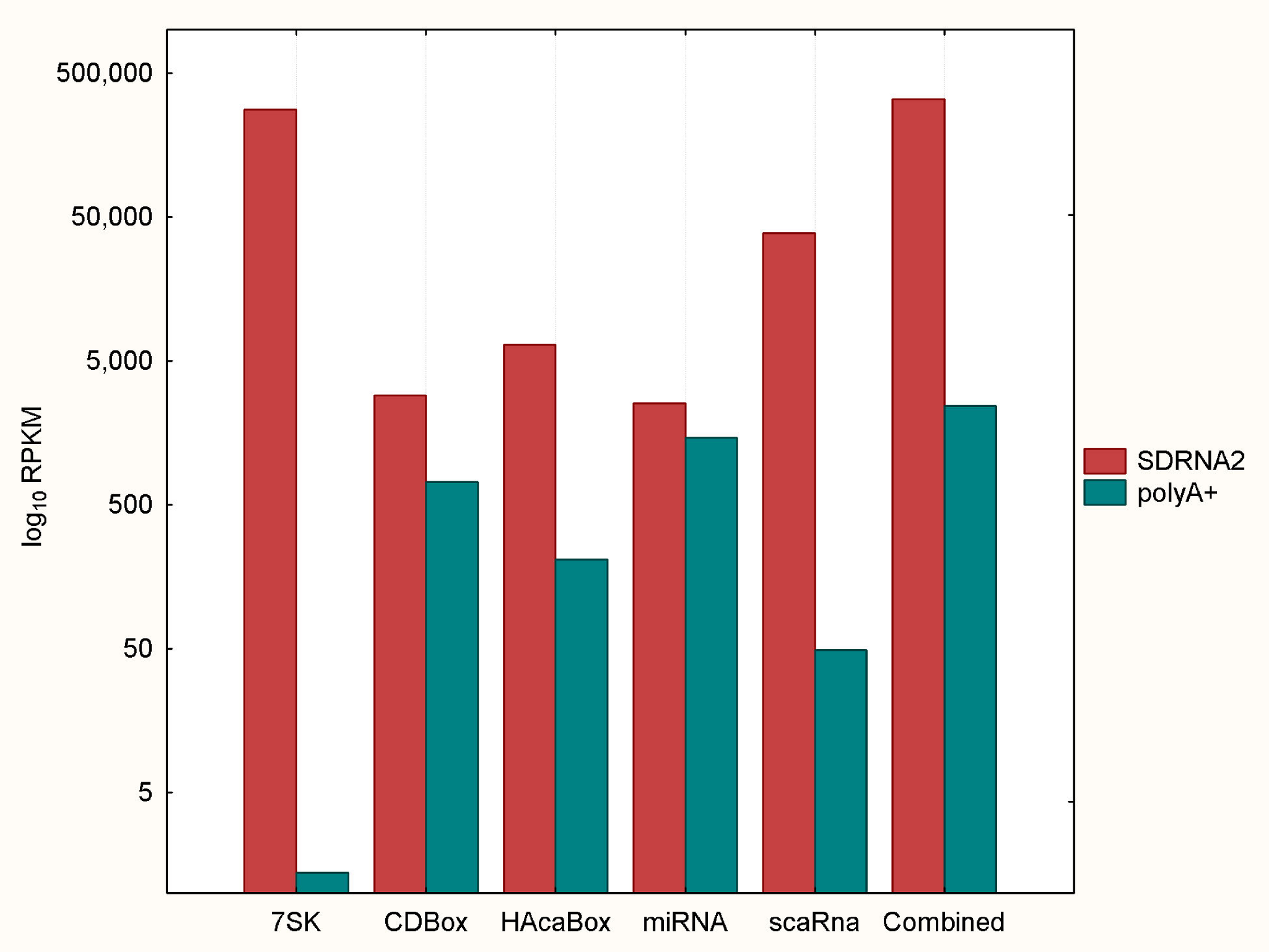

Supplement: Figure S2 — Enrichment of ncRNA transcripts in SDRNA libraries. Comparing FF breast depleted with SDRNA2 or polyA+ selection. Classes are based on UCSC Genome Browser annotations for sno/miRNAs. 7SK coordinates are from ENSEMBLE. (TIF) [file pone.0042882.s002.tif]

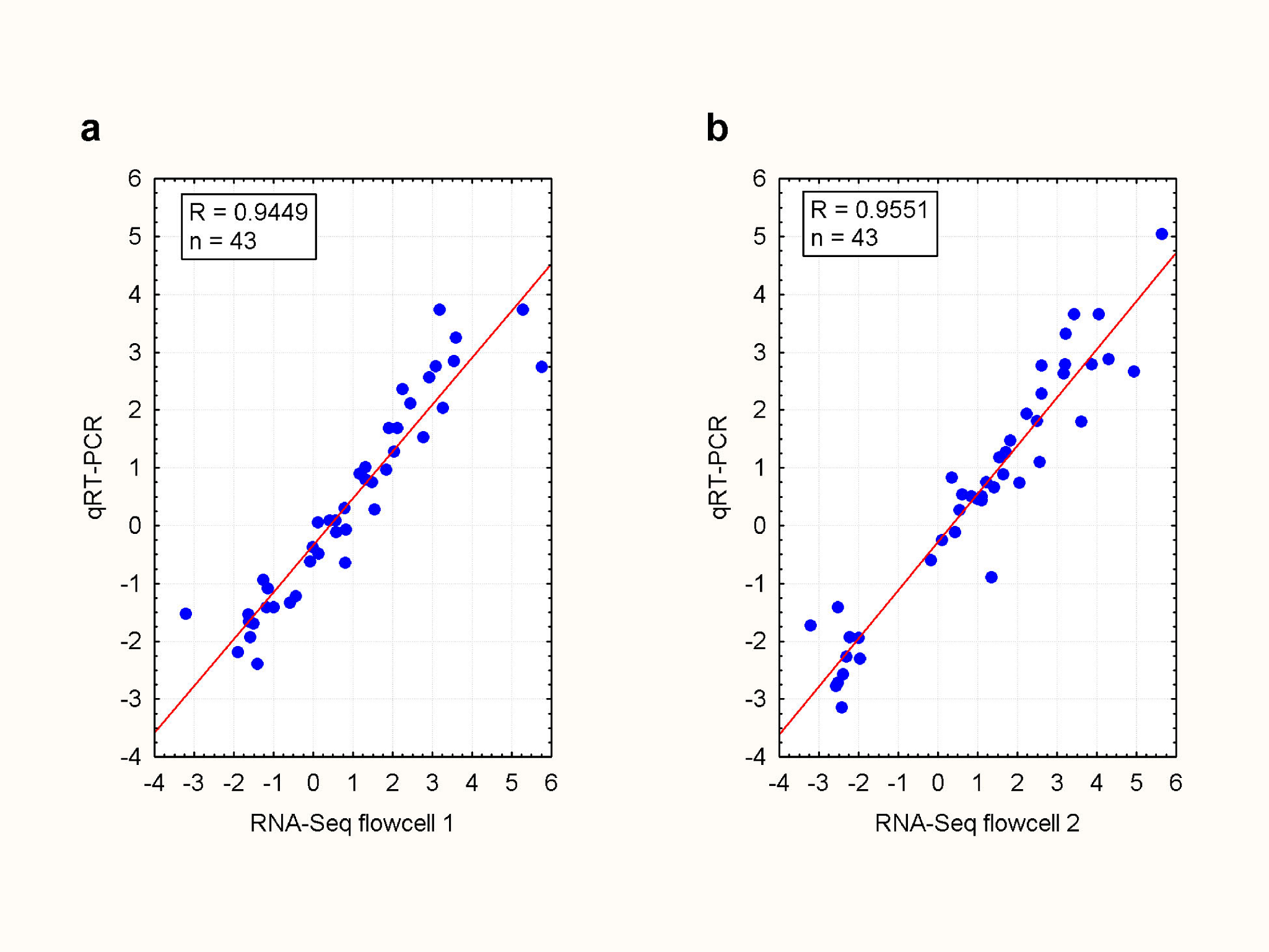

Supplement: Figure S3 — Cross-platform correlation of RefSeq transcript abundances. Twelve FFPE tumor tissue and twelve FFPE normal tissue breast cancer libraries were prepared using SDRNA1 and analyzed in sixteen lanes on two flowcells using indexing. Read abundance was averaged on each flowcell for each gene across all six tumor libraries and likewise across all six normal libraries. Read counts less than ten were excluded. Log2 Tumor/Normal fold-changes for forty-three genes are plotted between RNA-Seq data (x-axis) and TaqMan® RT-PCR data (y-axis). Scatterplots of average tumor/normal expression from (a) flowcell 1 and (b) flowcell 2. (TIF) [file pone.0042882.s003.tif]
